# Effect of theaflavin-3,3′-digallate on leptin-deficient induced nonalcoholic fatty liver disease might be related to lipid metabolism regulated by the Fads1/PPARδ/Fabp4 axis and gut microbiota

**DOI:** 10.3389/fphar.2022.925264

**Published:** 2022-08-29

**Authors:** Cheng Zhou, Wenji Zhang, Hui Lin, Luyun Zhang, Fan Wu, Yan Wang, Susu Yu, Xinyue Peng, Wenli Cheng, Min Li, Xiaoying Pan, Zhenrui Huang, Wenjuan Zhang

**Affiliations:** ^1^ Department of Public Health and Preventive Medicine, School of Medicine, Jinan University, Guangzhou, China; ^2^ Guangdong Provincial Engineering & Technology Research Center for Tobacco Breeding and Comprehensive Utilization, Key Laboratory of Crop Genetic Improvement of Guangdong Province, Crops Research Institute, Guangdong Academy of Agricultural Sciences, Guangzhou, China; ^3^ Department of Radiation Oncology, Guangdong Provincial People’s Hospital, Guangdong Academy of Medical Sciences, Guangzhou, China

**Keywords:** TF3, nonalcoholic fatty liver disease, RNA sequencing, 16S rRNA, lipid metabolism

## Abstract

Nonalcoholic fatty liver disease (NAFLD), one of the risk factors for hepatitis, cirrhosis, and even hepatic carcinoma, has been a global public health problem. The polyphenol compound theaflavin-3,3′-digallate (TF3), mainly extracted from black tea, has been reported to produce an effect on hypoglycemic and antilipid deposition *in vitro*. In our study, we further investigated the function and novel mechanisms of TF3 in protecting NAFLD *in vivo*. By using leptin-deficient obese (ob/ob) mice with NAFLD symptoms, TF3 treatment prevented body weight and waistline gain, reduced lipid accumulation, and alleviated liver function injury, as well as decreased serum lipid levels and TG levels in livers in ob/ob mice, observing no side effects. Furthermore, the transcriptome sequencing of liver tissue showed that TF3 treatment corrected the expression profiles of livers in ob/ob mice compared with that of the model group. It is interesting to note that TF3 might regulate lipid metabolism *via* the Fads1/PPARδ/Fabp4 axis. In addition, 16S rRNA sequencing demonstrated that TF3 increased the abundance of *Prevotellaceae_UCG-001*, *norank_f_Ruminococcaceae*, and *GCA-900066575* and significantly decreased that of *Parvibacter.* Taken together, the effect of TF3 on NAFLD might be related to lipid metabolism regulated by the Fads1/PPARδ/Fabp4 axis and gut microbiota. TF3 might be a promising candidate for NAFLD therapy.

## Introduction

Nonalcoholic fatty liver disease (NAFLD), also called metabolic-associated fatty liver disease (MAFLD) (Eslam et al., 2020), affected approximately one quarter of the global adult population (Younossi et al., 2018). Considering the increasing morbidity of obesity and diabetes (Cotter and Rinella, 2020), the incidence of NAFLD has been increasing in recent years, and metabolic diseases were associated with greater NAFLD risk (Perumpail et al., 2017), which means NAFLD could place a heavier economic burden on health in the global societies. As a common chronic liver disease, NAFLD has a broad spectrum of clinical manifestations; simple steatosis could progress into nonalcoholic steatohepatitis, fibrosis, cirrhosis, and hepatocellular carcinoma (Manne et al., 2018; Liu et al., 2022). As the most widely prescribed lipid-lowering drugs, statins can be safely used to treat dyslipidemia in patients with NAFLD/NASH (Easl Easd And Easo, 2016; Chalasani et al., 2018). Their limited use is mainly attributed to hepatotoxicity such as asymptomatic raised aminotransferases (Nascimbeni et al., 2019), along with myalgia, hemorrhagic stroke, cognitive decline, peripheral neuropathy, insomnia, cataract, etc. (Mancini et al., 2011). Although trial evidence supports the efficacy of some diabetes drugs in patients with NAFLD or NASH, pioglitazone (Sanyal et al., 2010) might significantly increase weight, and metformin (Anushiravani et al., 2019) had no substantial impact on liver disease. At present, there is no specific treatment for NAFLD in clinic; thus, the effective drugs need to be actively explored.

Theaflavin-3,3′-digallate (TF3) was formed from the oxidation of selected pairs of catechins during tea processing and was one of the polyphenols in black tea. TF3, together with theaflavin (TF1), theaflavin-3-gallate (TF2A), theaflavin-3′-gallate (TF2B), belongs to the theaflavin (TF) category. They have the beneficial health effects and pharmacological activities (Wu et al., 2011; Fatima et al., 2013). As a more active monomer, TF3 has anticancer (Gao et al., 2019), cellular antioxidant (Jiang et al., 2021), and antibacterial (Wang S. et al., 2019) biological activities. Oral administration of a TF3-rich complex was reported to significantly decrease the adiposity index, enhance the insulin-sensitive index, inhibit the hepatic lipase activity, and slightly reduce leptin levels in livers in an obese Sprague-Dawley rat model (Jin et al., 2013). At a recent time, the effect of TFs extracted and purified from black tea was studied in high-fat diet–induced obese mice, and the results demonstrated that gavage administration of TFs might exert antihyperglycemic and lipid-lowering effects by inhibiting the synthesis and accumulation of lipids in the liver with activation of related pathways. Compared to other monomers of theaflavins, TF3 was proved to be the best choice (Cai et al., 2021). Our previous research *in vitro* also reported that TF3 is the best one among the theaflavin constituents in alleviating hepatocyte lipid deposition through activating an AMPK signaling pathway by inhibiting plasma kallikrein (Zhang et al., 2020). However, as a polyhydroxylated polyphenol, TF3 can associate with surrounding water molecules, forming a large hydration shell around the TF3 molecule, which makes it difficult to utilize through the transcellular transport (Lambert and Yang, 2003). Some studies reported that TF3 had poor systematic bioavailability through gavage or oral administration (Mulder et al., 2001; Henning et al., 2006). Therefore, the intraperitoneal injection is a feasible direction to enhance the bioavailability in animal experiments.

Hence, in this study, the effects of TF3 on NAFLD were assessed in an ob/ob mouse model by intraperitoneal injection treatment to improve bioavailability and find the novel regulatory mechanism through the preliminary “gut–liver” interaction at the individual level. With the maturity of bioinformatics, transcriptome sequencing and 16S rRNA sequencing have been used here for gene expression analysis to reveal the overall biological characteristics. Our study would further explore TF3 as a promising natural drug to prevent and treat NAFLD.

## Materials and methods

### Mice and treatment

The 5 specific pathogen-free male C57BL/6J and 25 ob/ob mice (7 weeks old) (Beijing, SCXK 2019-0008) were purchased from Beijing Huafukang Biotechnology Co., Ltd. (Beijing, China). These mice were housed in a 12/12-h light and dark cycle at a constant temperature (22°C ± 2°C) and provided with standard chow diet and free water in Ruiye Model Animal Biotechnology Co., Ltd. (Guangzhou, China) (Guangdong, SYXK 2020-0218). After 7 days of adaptive feeding, C57BL/6J mice were assigned to the control group with saline (WT), and ob/ob mice were randomized into five groups (*n* = 5): the model group with saline (ob/ob), the positive group with polyene phosphatidyl choline of 3000 μL/kg body weight (ob/ob + PPC), low TF3 group with the dose of 5 mg/kg body weight (ob/ob + L-TF3), middle TF3 group with the dose of 10 mg/kg body weight (ob/ob + M-TF3), and high TF3 group with the dose of 20 mg/kg body weight (ob/ob + H-TF3). The intraperitoneal injection administration was performed every day for 4 weeks. The body weight and food intake of mice were recorded every day, and the waist circumference was measured every 3 days. At the end of the experiment, the stool samples were taken before all mice were sacrificed. The whole blood samples were from retro-orbital blood collection and left at room temperature for at least 30 min and then separated by centrifugation (2,500 rpm for 20 min) to obtain serum. A small portion of the freshly isolated and weighed liver was fixed in 4% paraformaldehyde, and the remaining liver and sera were frozen immediately in liquid nitrogen and then were stored at −80°C until they were to be used. The adipose tissue (epididymal, perirenal, subcutaneous, and brown fats) samples as well as other organs were also isolated, collected, weighed, and then stored at −80°C for further analysis.

### Biochemical and histopathological analyses

The biochemical indicators in serum were measured using reagent kits of triglyceride (TG) (A110-1-1), total cholesterol (TC) (A111-1-1), high-density lipoprotein cholesterol (HDL-c) (A112-1-1), low-density lipoprotein cholesterol (LDL-c) (A113-1-1), free fatty acids (FFA) (A042-2-1), alanine aminotransferase (ALT) (C009-2-1), and aspartate aminotransferase (AST) (C010-2-1) from Nanjing Jiancheng Bioengineering Institute (Nanjing, China). The TG levels in the liver were detected using the same reagent kit as serum. The fixed liver tissues were embedded in paraffin. Sections with a thickness of 5 μm were obtained and stained using hematoxylin and eosin (H&E). All sections were observed under a motorized multifunctional upright fluorescence microscope (DM6000B, Leica, Germany).

### Transcriptome sequencing and bioinformatics analysis

Total RNA was extracted from the mouse liver tissue samples, and the concentration and purity were detected using NanoDrop 2000. RNA integrity was detected using agarose gel electrophoresis, and RIN value was determined using Agilent 2100. The mRNA was isolated from total RNA through utilizing magnetic beads with Oligo (dT) to perform A-T base pairing with the ploy-A tail at the 3′ end of eukaryotic mRNA. Fragmentation buffer was added to screen out the short-sequence fragments of mRNA, that is, those approximately 300 bp. Then, first-strand cDNA and second-strand cDNA were sequentially synthesized. The Illumina Novaseq 6000 System was used for sequencing. Before sequencing, the library was enriched, 15 cycles of PCR were used for amplification, and 2% agarose gels were used to recover the target bands. A TBS380 Mini-Fluorometer was used for quantification. Clusters were generated by bridge PCR amplification on a cBot System (Illumina). The transcriptome information was analyzed on the online platform of Majorbio Cloud Platform (www.majorbio.com). To identify the differentially expressed genes (DEGs) of liver tissues, those that had Fold Change ≥ 1.5 and adjusted *p* < 0.05 were considered statistically significant. Gene Ontology (GO) and Kyoto Encyclopedia of Genes and Genomes (KEGG) enrichment analyses were performed to explore the biological functions of the DEGs and pathways they enriched significantly. The Benjamini–Hochberg (B-H) multiple test correction method was used to correct the false positives (adjusted *p* < 0.05).

### 16S rRNA sequencing and bioinformatics analysis

Total community DNA extracted from the stool samples of mice was used for PCR amplification. Then, PCR amplification products were detected and quantified using the QuantiFluor-ST™ blue fluorescence quantification system (Promega). The purified amplicons were combined in equimolar masses and sequenced on the Illumina MiSeq PE300 platform (Illumina, San Diego, United States). After subsampling each sample to an equal sequencing depth (39,800 reads per sample) and clustering for the next analysis, the alpha diversities of the gut microbiota in samples were calculated using the observed richness (sobs) and the diversity (shannon) index. The Good’s coverage and rarefaction curves were used to determine whether the sequencing amount was sufficient. Beta diversity was determined using the OTUs from each sample, and the similarity between samples was calculated using the unweighted unifrac and represented in principal co-ordinate analysis (PCoA). Welch’s *t* test was performed to obtain species with significant differences between two groups. The data of 16S rRNA sequencing were also analyzed on the online platform of Majorbio Cloud Platform (www.majorbio.com).

### Statistical analysis

The data were presented as the mean ± standard deviation. Differential analysis was performed using SPSS 20.0 with the significance criterion set at *p* < 0.05. Student’s *t* test was used to assess the differences between two groups. One-way analysis of variance with Dunnett’s *post hoc* test and a nonparametric test were utilized for comparisons among multiple groups. Figures were generated using GraphPad Prism 8.3.0 or Majorbio Cloud Platform.

## Results

### Effect of theaflavin-3,3′-digallate on growth parameters and organ coefficients of nonalcoholic fatty liver disease in ob/ob mice

**FIGURE 1:**
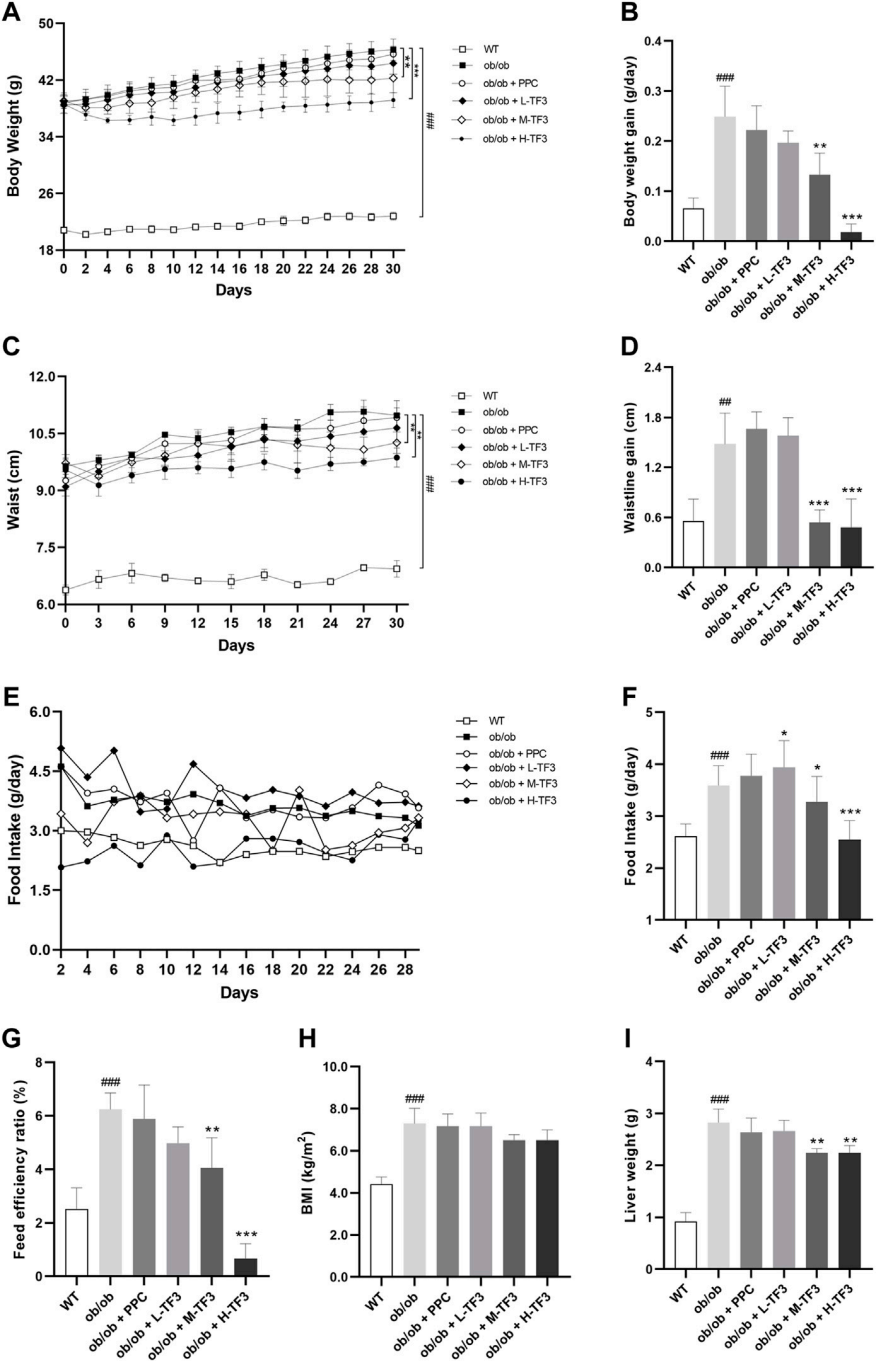
Body weight, waistline, food intake, BMI, and liver weight. **(A)** The trend of weight change within 4 weeks. **(B)** Body weight gain. **(C)** The trend of waistline changes within 4 weeks. **(D)** Waistline gain. **(E)** The trend of food intake changes within 4 weeks. **(F)** Average daily food intake. **(G)** Feed efficiency ratio. Feed efficiency ratio (%) = body weight gain (g/day)/food intake (g/day) × 100%. **(H)** BMI. **(I)** Liver weight. All data are shown as the mean or mean ± standard deviation (*n* = 5). Statistical significance: ^#^ *p* < 0.05, ^##^ *p* < 0.01, ^###^ *p* < 0.001 vs. the WT group. ^*^ *p* < 0.05, ^**^ *p* < 0.01, ^***^ *p* < 0.001 vs. the ob/ob group.

**FIGURE 2:**
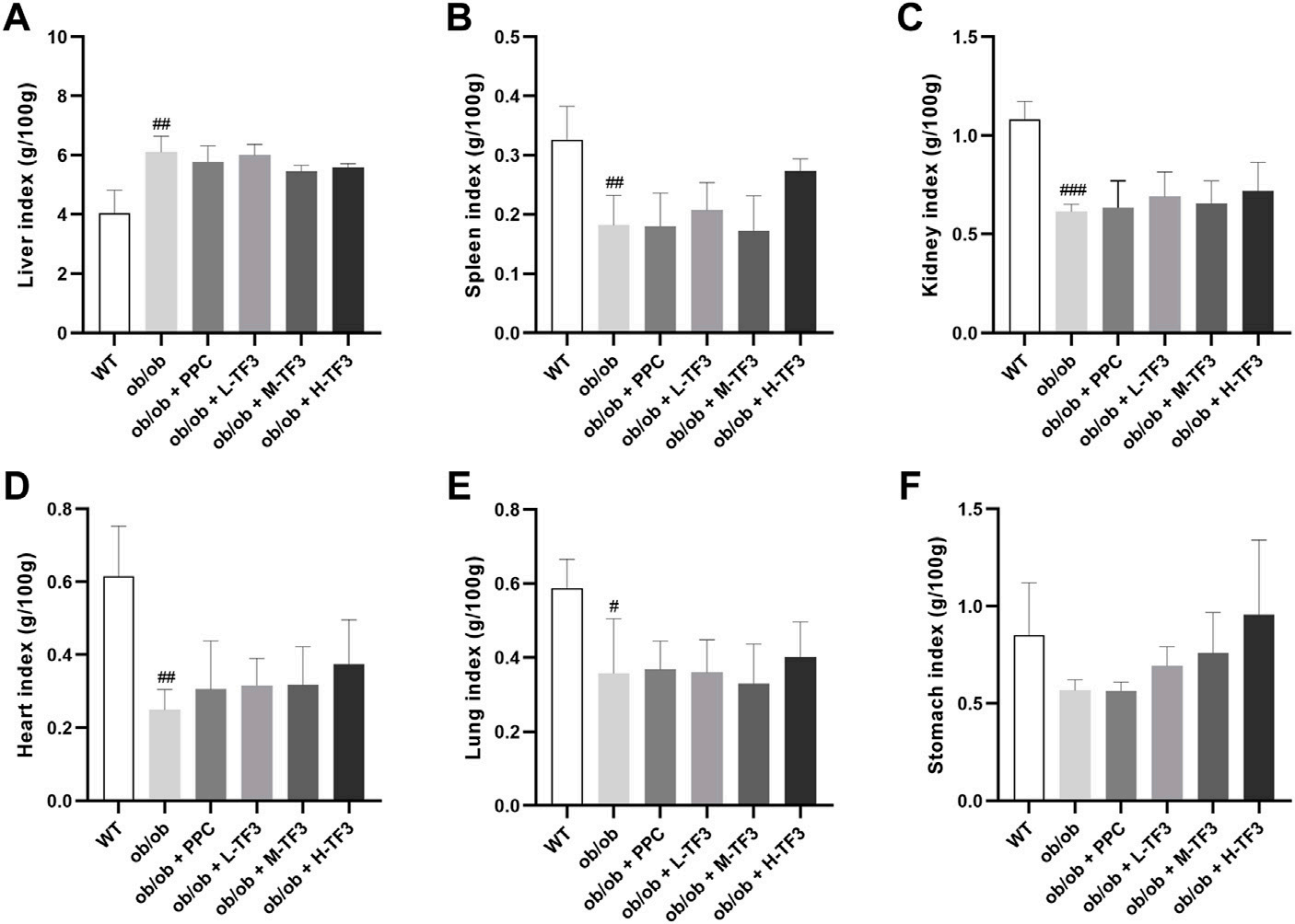
Organ coefficients. **(A)** Liver index. **(B)** Spleen index. **(C)** Kidney index. **(D)** Heart index. **(E)** Lung index. **(F)** Stomach index. All data are shown as the mean ± standard deviation (*n* = 5). Statistical significance: ^#^ *p* < 0.05, ^##^ *p* < 0.01, ^###^ *p* < 0.001 vs. the WT group.

To observe the effect of TF3 on NAFLD, we found that the final body weight and waistline, that is, body weight gain and waistline gain, of ob/ob mice in the model group both were higher than those of mice in the control group (*p* < 0.01 or *p* < 0.001). In comparison with those in the model group, these growth parameters all significantly reduced in the M-TF3 and H-TF3 groups with *p* values less than 0.01 or 0.001, respectively (Figures 1A–D). In particular, body weight gain in the H-TF3 group was only 1/14 of that in the ob/ob group. In Figures 1E, F, significant differences were observed in the average daily food intake between all the TF3 groups and the ob/ob group. We find it interesting that L-TF3 treatment made food intake increased (*p* < 0.05), while M-TF3 and H-TF3 treatment decreased food intake (*p* < 0.05 and *p* < 0.001, respectively). Moreover, the feed efficiency ratio declined from 6.2 to 0.7% in ob/ob mice with H-TF3 treatment (*p* < 0.001). The M-TF3 groups also exhibited a decrease in feed efficiency ratio compared with the ob/ob group (*p* < 0.01) (Figure 1G). In contrast, the BMI was not observed to have a significant change in the TF3 groups and ob/ob group from Figure 1H (*p* > 0.05). Liver weight in the M-TF3 and H-TF3 groups was lighter than that in the ob/ob group (*p* < 0.01) (Figure 1I). The organ coefficients showed no significant difference in all ob/ob mice (*p* > 0.05) (Figures 2A–F). The viability of mice was not affected, except that the mice in the H-TF3 group had slightly less smooth hair during adaptation within the first 3 days of administration, but it recovered quickly.

### Theaflavin-3,3′-digallate decreased fat accumulation of nonalcoholic fatty liver disease in ob/ob mice

**FIGURE 3:**
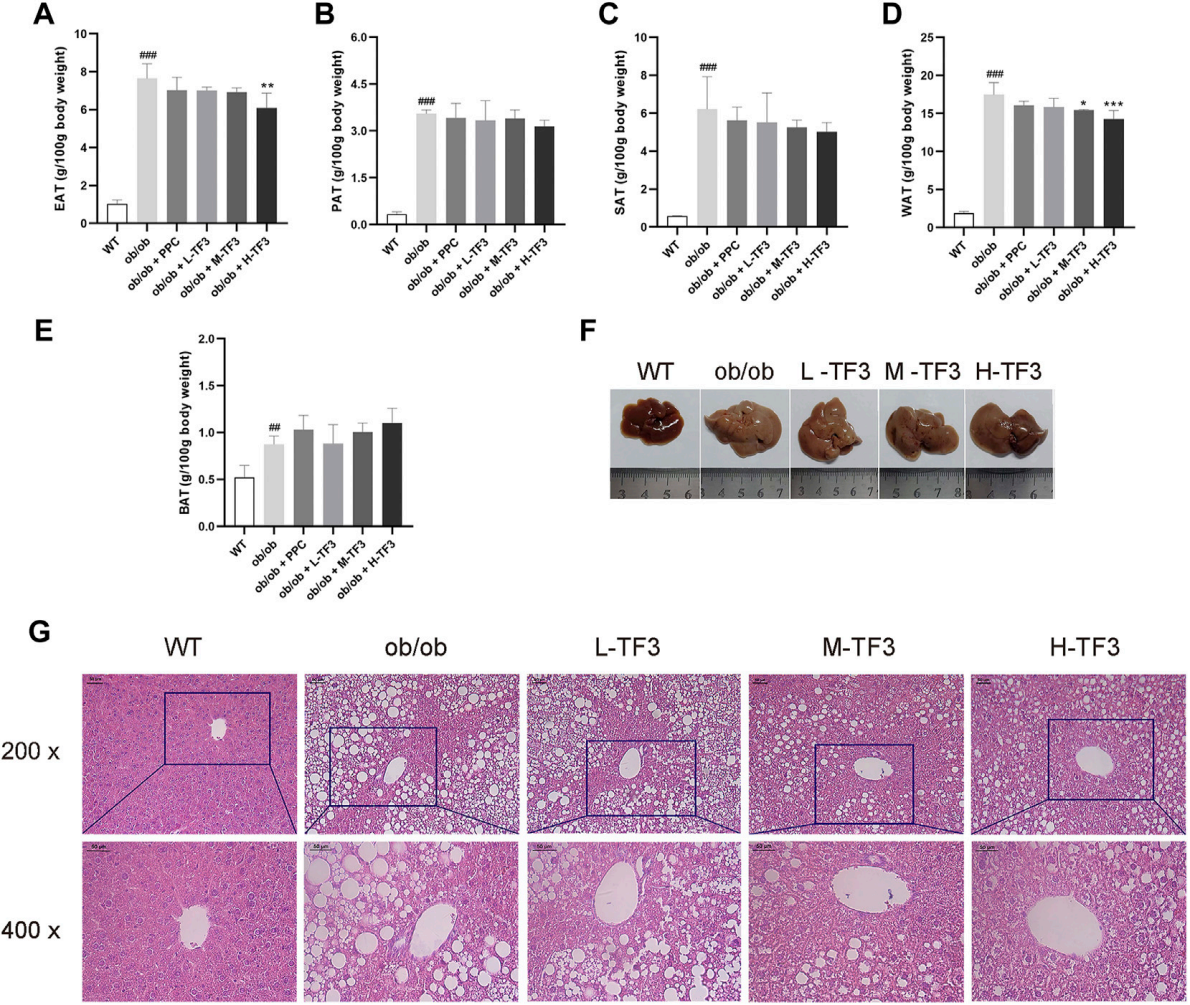
Weight of adipose tissue, hepatic appearance, and hematoxylin and eosin (H&E) staining. **(A)** Weight of epididymal adipose tissue. **(B)** Weight of subcutaneous adipose tissue. **(C)** Weight of perineal adipose tissue. **(D)** Weight of white adipose tissue; white adipose tissue was the sum of subcutaneous, perineal, and epididymal adipose tissue. **(E)** Weight of brown adipose tissue. **(F)** Liver appearance. **(G)** The representative image of the liver slides in H&E staining (scale bar, 50 µm for ×200 and ×400 magnification). All data are shown as the mean ± standard deviation (*n* = 5). Statistical significance: ^#^ *p* < 0.05, ^##^ *p* < 0.01, ^###^ *p* < 0.001 vs. the WT group. ^*^ *p* < 0.05, ^**^ *p* < 0.01, ^***^ *p* < 0.001 vs. the ob/ob group.

Although the epididymal adipose tissue (EAT), subcutaneous adipose tissue (SAT), perirenal adipose tissue (PAT), and brown adipose tissue had been weighted in the experimental groups and model group, the relative weight of EAT in the H-TF3 group was significantly lower than that in the ob/ob group with *p* < 0.01, and white adipose tissue (WAT, the sum of EAT, SAT, and PAT) (g/100 g body weight) decreased in the M-TF3 and H-TF3 groups with *p* < 0.05 and *p* < 0.001, respectively (Figures 3A–E).

### Theaflavin-3,3′-digallate attenuated liver tissue variation of nonalcoholic fatty liver disease in ob/ob mice

Liver size in ob/ob mice dramatically increased with a much lighter color than that in wild mice, which might indicate a higher fat content. The mice in the M-TF3 and H-TF3 groups were seen with obviously smaller liver size than that of the ob/ob group. These changes were improved sequentially as the dose of TF3 increases. M-TF3 and H-TF3 treatment made liver size smaller and deepened the color (Figure 3F). H&E staining of pathological slice observation showed prominent diffuse hepatic steatosis with nuclear condensation, cytoplasmic looseness, and increased fat vacuoles in ob/ob mice compared to wild mice. M-TF3 and H-TF3 treatment significantly reversed these changes (Figure 3G). Taken together, TF3 changed the appearance of the liver and alleviated hepatic steatosis in ob/ob mice.

### Theaflavin-3,3′-digallate reduced blood lipid, liver function injury, and hepatic triglyceride of nonalcoholic fatty liver disease in ob/ob mice

**FIGURE 4:**
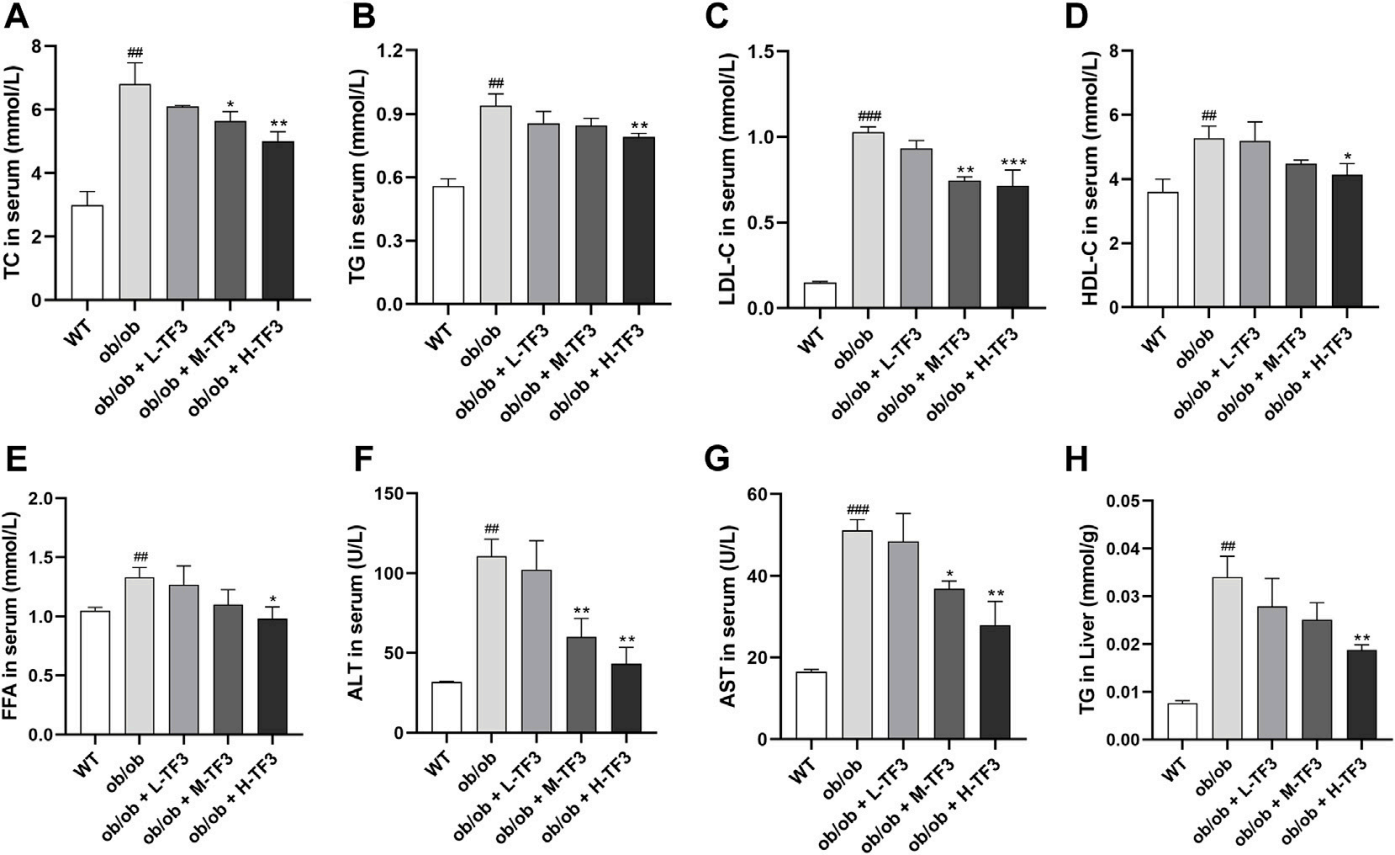
Biochemical parameters. **(A)** Total cholesterol (TC) level in serum. **(B)** Triglyceride (TG) level in serum. **(C)** Low-density lipoprotein cholesterol (LDL-c) level in serum. **(D)** High-density lipoprotein cholesterol (HDL-c) level in serum. **(E)** Free fatty acid (FFA) level in serum. **(F)** Alanine aminotransferase (ALT) level in serum. **(G)** Aspartate aminotransferase (AST) level in serum. **(H)** TG level in liver. Each parameter was repeated three times independently. All data are shown as the standard deviation (*n* = 3). Statistical significance: ^#^ *p* < 0.05, ^##^ *p* < 0.01, ^###^ *p* < 0.001 vs. the WT group. ^*^ *p* < 0.05, ^**^ *p* < 0.01, ^***^ *p* < 0.001 vs. the ob/ob group.

TF3 significantly decreased the levels of TC and LDL-c in the M-TF3 and H-TF3 treatment group compared to the ob/ob group. TC (*p* < 0.01) and LDL-c (*p* < 0.001) in H-TF3 mice were lower than TC (*p* < 0.05) and LDL-c (*p* < 0.01) in M-TF3 mice. In contrast, obvious difference in TG (*p* < 0.01), HDL-c (*p* < 0.05), and FFA (*p* < 0.05) were observed only by H-TF3 supplementation compared with the ob/ob mice (Figures 4A–E). The elevated ALT and AST were also significantly decreased in the M-TF3 and H-TF3 intervention group compared to those in the ob/ob model group with dose-dependent trends (Figures 4F, G). AST (*p* < 0.05) and ALT (*p* < 0.01) in the M-TF3 group and AST (*p* < 0.01) and ALT (*p* < 0.01) in the H-TF3 group were 28.0% and 45.7% and 45.4% and 60.9% lower than those in the ob/ob group, respectively. In particular, the level of ALT in the H-TF3 group was close to that in the normal wild mice. H-TF3 treatment also reversed the elevated hepatic TG (*p* < 0.01) level induced by ob/ob obese mice (Figure 4H).

### Theaflavin-3,3′-digallate regulated the hepatic gene expression profile

**FIGURE 5:**
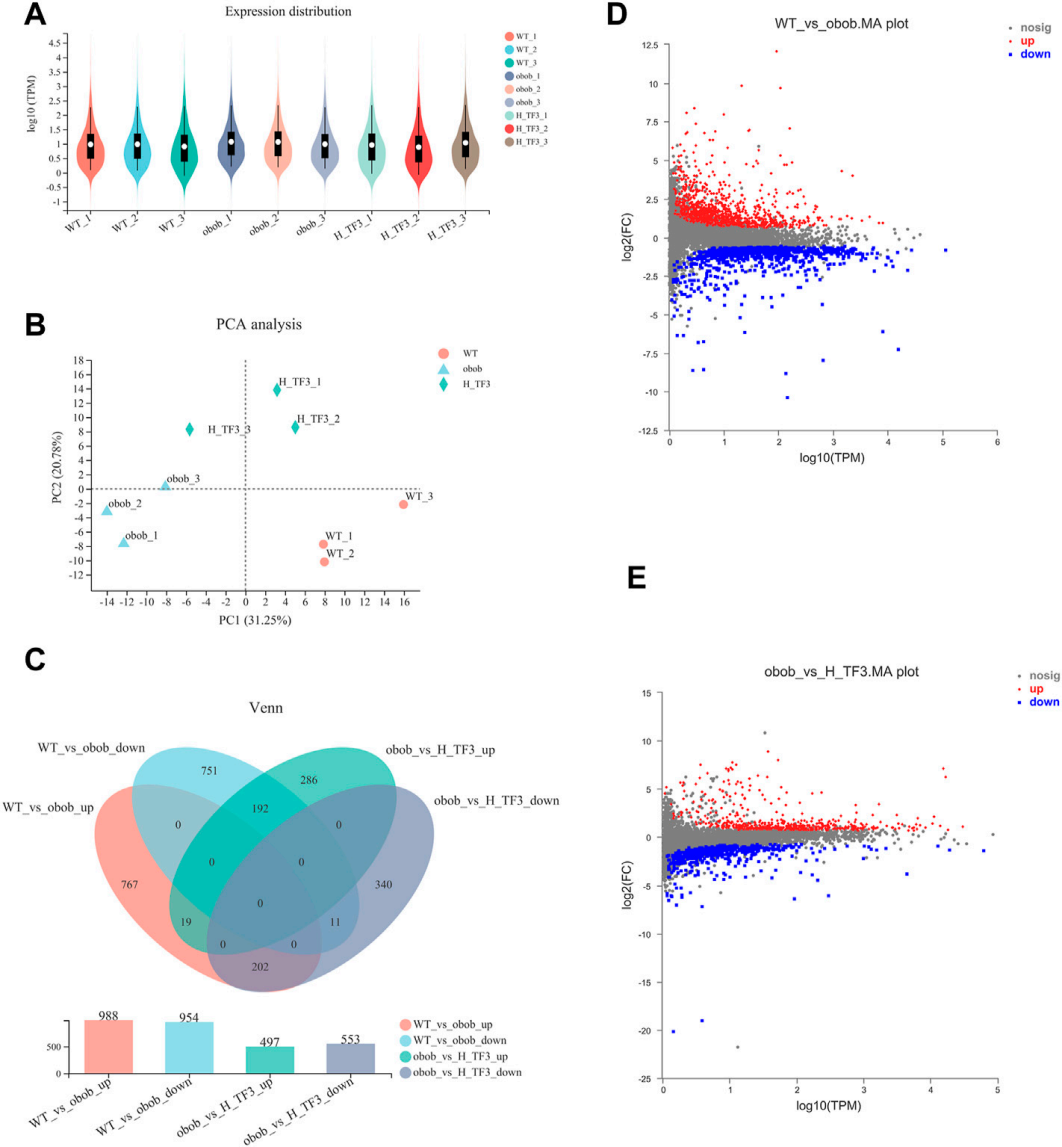
Comparison of gene expression (*n* = 3). **(A)** Violin plot showed gene expression distribution of each sample. **(B)** Principal component analysis (PCA) of gene expression profiles of three groups in liver tissue. **(C)** Venn diagram summarized gene changes in expression in various gene sets. **(D)** MA plot represented upregulated and downregulated genes in the WT vs. ob/ob comparison group. **(E)** MA plot represented upregulated and downregulated genes in the ob/ob vs. H-TF3 comparison group.

Based on the sequencing results of the WT, ob/ob, and H-TF3 groups, the sequencing quality was high and the sequencing depth was sufficient for transcriptome analysis (Supplementary Table S1). The expression distribution (Figure 5A) and principal component analysis (Figure 5B) indicated that biological reproducibility between samples was enough to subsequent analysis. Differential expression analysis and a Venn plot showed that there were 1,942 DEGs in the WT vs. ob/ob comparison including 988 upregulated DEGs and 954 downregulated DEGs. Moreover, 1,050 DEGs included 497 upregulated DEGs and 553 downregulated DEGs in the ob/ob vs. H-TF3 comparison (Figure 5C). These DEGs were subjected to additional bioinformatics analysis by being created as target gene sets. The MA plots showed the distribution of upregulated and downregulated DEGs in two comparison groups (Figures 5D, E).

**FIGURE 6:**
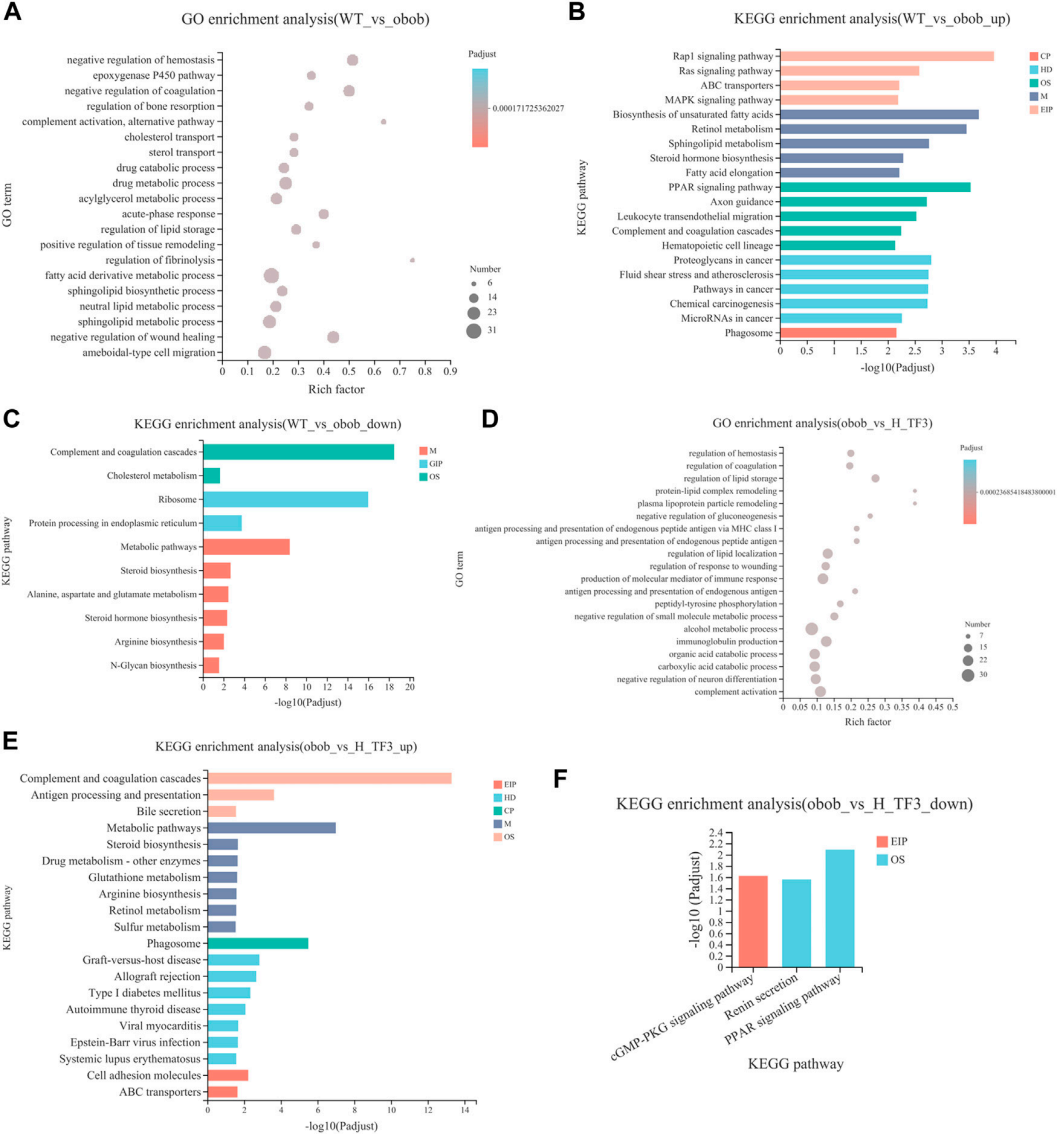
GO and KEGG enrichment analyses of DEGs between WT vs. ob/ob and ob/ob vs. H-TF3 comparison group (*n* = 3). **(A)** Top 20 enriched GO terms from DEGs in the WT vs. ob/ob group. **(B)** Top 20 enriched KEGG pathways from upregulated DEGs in the WT vs. ob/ob group. **(C)** Enriched KEGG pathways from downregulated DEGs in the WT vs. ob/ob group. **(D)** Top 20 enriched GO terms from DEGs in the ob/ob vs. H-TF3 group. **(E)** Top 20 enriched KEGG pathways from upregulated DEGs in the ob/ob vs. H-TF3 group. **(F)** Enriched KEGG pathways from downregulated DEGs in the ob/ob vs. H-TF3 group.

GO and KEGG enrichment analyses were performed in target gene sets. GO terms with the top 20 enrichment degrees showed that the cholesterol transport, sterol transport, regulation of lipid storage, and fatty acid derivative metabolic process were closely related to NAFLD in obese mice (Figure 6A). KEGG enrichment analysis showed that the upregulated DEGs of the WT and ob/ob groups were mainly enriched in pathways of fatty acid elongation and steroid hormone biosynthesis, fatty acid metabolism, metabolic pathways, peroxisome proliferator-activated receptor (PPAR) signaling pathway, and biosynthesis of unsaturated fatty acids pathways (Figure 6B). In addition, the downregulated DEGs were enriched in steroid hormone biosynthesis, metabolic pathways, and cholesterol metabolism (Figure 6C). In the ob/ob and H-TF3 groups, the DEGs were primarily distributed in the pathways of regulation of lipid storage, secondary alcohol biosynthetic process, energy reserve metabolic process, and steroid biosynthetic and metabolic process (Figure 6D). KEGG enrichment analysis showed that downregulated DEGs between the ob/ob group and the H-TF3 group were mainly enriched in the PPAR signaling pathway (Figure 6E), while upregulated DEGs were mostly distributed in metabolic pathways, type I diabetes mellitus, steroid biosynthesis, bile secretion, etc. (Figure 6F). These results suggested that metabolic pathways and the PPAR signaling pathway might be crucial pathways producing an effect on NAFLD in this study. Moreover, 77 DEGs of metabolic pathways in the ob/ob vs. H-TF3 group were selected to create a histogram of KEGG analysis. It is interesting to note that they were mainly annotated to the lipid metabolism process including biosynthesis of unsaturated fatty acids (Fads1, Tecr, Scd1, and Elovl1), arachidonic acid and linoleic acid metabolism (Cyp4f14, Cyp1a2, and Cyp2c70), and steroid biosynthesis (Fdft1, Tm7sf2, Ebp, Dhcr7). These genes were significantly upregulated in ob/ob mice with TF3 treatment. In the PPAR signaling pathway, the expression of Fabp4, Plin4, Lpl, and Acadm was decreased, while that of Ppard encoding peroxisome proliferator-activated receptors δ (PPARδ) was increased. Based on the above results, we supposed that TF3 treatment might alleviate NAFLD through lipid metabolism regulated by the Fads1/PPARδ/Fabp4 axis.

### Theaflavin-3,3′-digallate altered gut microbiota structure

**FIGURE 7:**
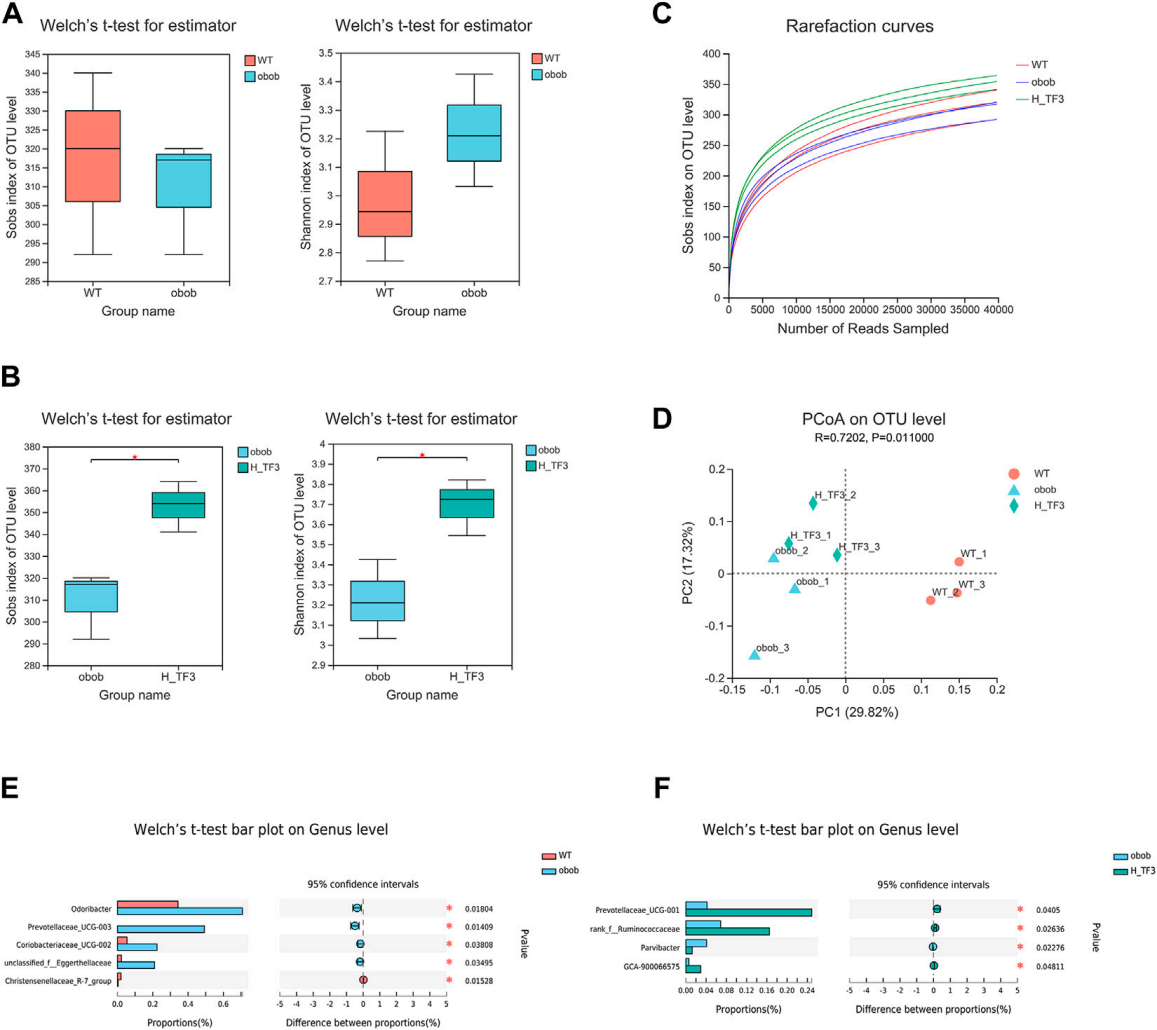
Different gut microbiota composition in the WT vs. ob/ob group and the ob/ob vs. H-TF3 comparison group (*n* = 3). **(A)** Alpha diversity index (Sobs and Shannon) compared between the WT and ob/ob groups. **(B)** Alpha diversity index (Sobs and Shannon) compared between the ob/ob and H-TF3 groups. **(C)** Rarefaction curves. **(D)** Beta diversity analysis. Principal coordinates analysis (PCoA) based on unweighted unifrac at the OTU level among the three groups. **(E)** Bar plot showed the different gut microbiota at the genus level in the WT and ob/ob groups using Welch’s *t* test. **(F)** Bar plot showed the different gut microbiota at the genus level in the ob/ob and H-TF3 groups using Welch’s *t* test. Statistical significance: ^*^ *p* < 0.05.

The results of 16S rRNA demonstrated that the Sobs and Shannon indices showed no significant difference between the WT and ob/ob groups. In ob/ob obese mice, no significant effect on the richness and diversity of the gut microbiota was observed compared to that in wild mice (Figure 7A). In contrast, the Sobs and Shannon indices were both significantly increased with H-TF3 treatment compared with those in the ob/ob mice group (Figure 7B), indicating that H-TF3 could enhance the richness and diversity of the gut microbiota. The Good’s coverage was up to 99% in each sample, and the rarefaction curves showed clear asymptotes (Figure 7C), which together indicated that the depth of sequencing data was sufficient and adequately covered most of the microbial diversity information in the sample. Moreover, PCoA revealed that the gut microbiota composition was different among the three groups (Figure 7D). To assess specific changes in the gut microbiota, we compared the relative abundance at the genus level. The relative abundance of *Odoribacter*, *Prevotellaceae_UCG-003*, *unclassified_f__Eggerthellaceae*, and *Prevotellaceae_Ga6A1_group* was significantly increased, whereas the relative abundance of *Christensenellaceae_R-7_group* and *Ruminococcus* was decreased in that ob/ob group compared with those in the WT group (*p* < 0.05) (Figure 7E). In contrast, the relative abundance of *Prevotellaceae_UCG-001*, *norank_f__Ruminococcaceae*, and *GCA-900066575* was significantly increased, and that of *Parvibacter* was significantly decreased in the H-TF3 group compared with those in the ob/ob group (*p* < 0.05) (Figure 7F). Gut microbiota in ob/ob mice differentiated from that of WT group mice. TF3 treatment changed gut microbiota composition in ob/ob mice.

### Associations between the gut microbiota composition and biochemical indicators and candidate genes

**FIGURE 8:**
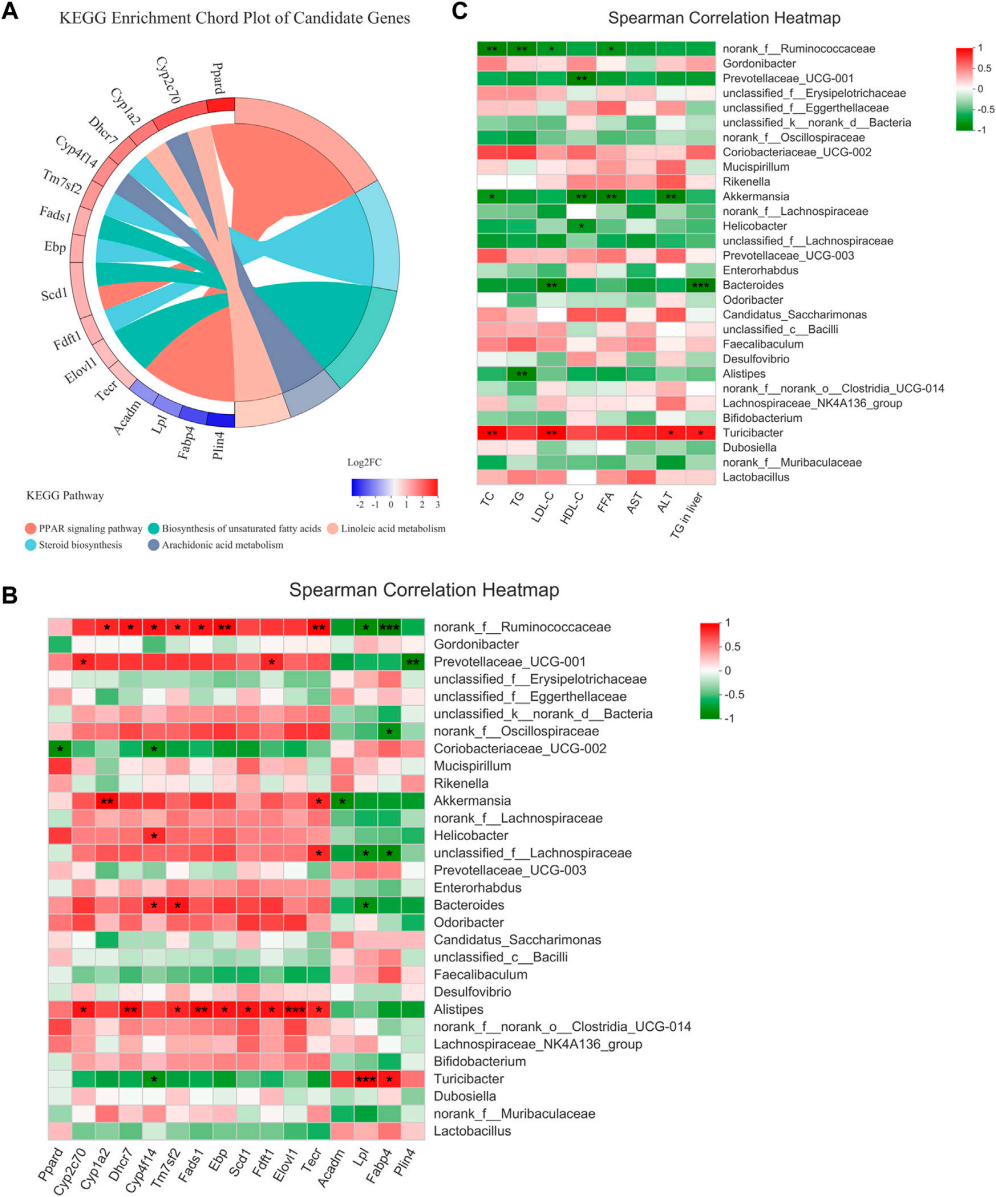
Candidate genes and Spearman correlation heatmap (*n* = 3). **(A)** KEGG enrichment chord plot of candidate genes. Left of plot is the regulation and fold change of expression; right is KEGG pathways. **(B)** The correlation between the gut microbiota and candidate genes. **(C)** The correlation between the gut microbiota and serum biochemical parameters. Statistical significance: ^*^ *p* < 0.05, ^**^ *p* < 0.01.

The expression of the 11 DEGs involved in lipid metabolism and 5 DEGs in the PPAR signaling pathway were reversed by H-TF3 treatment in ob/ob mice with significant difference (adjusted *p* < 0.05). Their differences in expression are shown in Figure 8A and Table 1. For further correlation analysis, 16 DEGs served as candidate genes that potentially functioned after TF3 treatment in NAFLD. The correlation between liver candidate genes and gut microbiota are shown in a Spearman correlation heatmap (Figure 8B). The relative abundance of *norank_f__Ruminococcaceae* genus exhibited a strong correlation with the expression of Cyp1a2, Dhcr7, Cyp4f14, Tm7sf2, Fads1, Ebp, Scd1, Tecr, Lpl, and Fabp4 (*p* < 0.05), especially those genes involved in the Fads1/PPARδ/Fabp4 signaling axis. We find it interesting that the relative abundance of *Alistipes* also showed a significantly positive correlation with the expression of some candidate genes, including Cyp2c70, Dhcr7, Tm7sf2, Fads1, Ebp, Scd1, Fdft1, Elovl1, and Tecr (*p* < 0.05). In addition, the correlation between biochemical parameters and intestinal flora are shown in a Spearman correlation heatmap (Figure 8C). The relative abundance of *norank_f__Ruminococcaceae* exhibited a significantly negative correlation with serum TC, TG, LDL-c, and FFA (*p* < 0.05). The relative abundance of *Akkermansia* was negatively correlated with the level of TC, HDL-c, FFA, and ALT in serum (*p* < 0.05). Preliminary correlation analyses demonstrated that TF3 might have a multidimensional effect on NAFLD mitigation.

**TABLE 1:** List of candidate genes (obob_vs._H_TF3).

Gene name	Gene description	Regulation
Ppard	peroxisome proliferator activator receptor delta	up
Cyp2c70	cytochrome P450, family 2, subfamily c, polypeptide 70	up
Cyp1a2	cytochrome P450, family 1, subfamily a, polypeptide 2	up
Plin4	perilipin 4	down
Dhcr7	7-dehydrocholesterol reductase	up
Fabp4	fatty acid binding protein 4, adipocyte	down
Cyp4f14	cytochrome P450, family 4, subfamily f, polypeptide 14	up
Tm7sf2	transmembrane 7 superfamily member 2	up
Lpl	lipoprotein lipase	down
Fdft1	farnesyl diphosphate farnesyl transferase 1	up
Ebp	phenylalkylamine Ca^2+^ antagonist (emopamil) binding protein	up
Fads1	fatty acid desaturase 1	up
Tecr	trans-2,3-enoyl-CoA reductase	up
Scd1	stearoyl-Coenzyme A desaturase 1	up
Acadm	acyl-Coenzyme A dehydrogenase, medium chain	down
Elovl1	elongation of very long chain fatty acids	up

## Discussion

The lipid-lowering effects of theaflavin monomer TF3 have been authenticated *in vitro and vivo* in the recent 5 years, although there are only a few studies. Our previous study first confirmed that TF3 relieved hepatocyte lipid deposition through the novel plasma kallikrein/AMPK signaling pathway in FFA-induced hepatic HepG2 cells (Zhang et al., 2020). A recent study reported that TF3 could ameliorate obesity in high-fat diet–induced mice by oral administration for 9 weeks through the signaling pathway of SIRT6/AMPK/SREBP-1/FASN. Even if the bioavailability of TF3 is considered poor, the beneficial effects were observed in the experimental design (Cai et al., 2021). In this NAFLD-related study, considering the lower bioavailability, the intraperitoneal injection administration of TF3 was applied to the ob/ob mouse model with more homogeneous phenotype. We found that TF3 rapidly produced beneficial effects based on the changes of weight and waistline in 1 week without bouncing until the end of the experiment. The results of the 4-week experiment gave us a reason to believe that TF3 has an obviously stable and effective therapeutic effect. Compared with oral administration for 9 weeks, intraperitoneal injection administration greatly shortened time and cost as well as improved the efficiency of animal experiment. Moreover, we found that the Fads1/PPARδ/Fabp4 signaling axis might be a new therapeutic target for NAFLD in the current study.

TF3 improved NAFLD in ob/ob mice. As expected, TF3 supplement decreased body weight, body weight gain, waistline, waistline gain in obese mice with food intake reduction. Effect of TF3 on phenotype was possibly related to suppression on appetite, which is in accord with the fact that black tea drinking makes people consume less food (Carter and Drewnowski, 2012). The organ coefficients were not obviously affected by TF3 treatment, indicating that TF3 is a relatively safe pharmacological ingredient. We could draw a conclusion of hepatic steatosis improving from histopathological analysis, which consisted of phenotypic changes in mice. The EAT with lighter weight was observed; it was an important organ for TG accumulation and many adipokines secretion (Corton et al., 1994). According to the results of biochemical indicators, TF3 had protective effects by decreasing the serum lipid and TG levels in the liver and could restore the activity of the liver function enzymes of AST and ALT.

The hepatic transcriptomics analysis indicated that metabolic pathways and the PPAR signaling pathway might be crucial pathways producing the effect of TF3. Metabolic pathways included biosynthesis of unsaturated fatty acids, arachidonic acid and linoleic acid metabolism, and steroid biosynthesis. The results are similar with the mechanism of turmeric in preventing hyperlipidemia in mice (Wang et al., 2021). Moreover, we proposed that TF3 produced an effect on NAFLD by regulating lipid metabolism *via* the Fads1/PPARδ/Fabp4 signaling axis. First, TF3 acted on upstream Fads1 and then activated PPARδ and downstream Fabp4. Fatty acid desaturase 1 encoded by Fads1 is one of the rate-limiting enzymes in the polyunsaturated fatty acid (PUFA) desaturation pathway (Martinelli et al., 2008). Patients with NAFLD had pathological changes associated with the depletion of PUFA; the higher expression of Fads1 protects liver from lipid accumulation (Araya et al., 2010; Athinarayanan et al., 2021). Moreover, Fads1 is specifically involved in catalyzing the conversion of dihomo-gamma-linolenic acid to arachidonic acid (Martinelli et al., 2008). Arachidonic acid and linoleic acid both belong to unsaturated fatty acids. Arachidonic acid is an important ω-6 PUFA, which is the most widely distributed endogenous active substance *in vivo*. A study reported that *Sagittaria sagittifolia* polysaccharide exerted preventive protection against high-fat diet–induced NAFLD by interfering with arachidonic acid metabolism (Deng et al., 2020). We find it interesting that the epoxyeicosatrienoic acids (EETs) are products of arachidonic acid and can be catalyzed by cytochrome P450 epoxygenases, in line with the increased expression of Cyp4f14, CYP1a2, and Cyp2c70 in this study. EETs and their metabolites can activate peroxisome proliferator-activated receptors α (PPARα) and peroxisome proliferator-activated receptors γ (PPARγ) in the PPAR signaling pathway (Wang X. et al., 2019). The PPAR signaling pathway is actively involved in the regulation of lipid metabolism, glucose homeostasis, cell proliferation, and adipocyte differentiation (Hu N. et al., 2021). The PPARδ is one of ligand-activated transcription factors that can be activated by the ligand of unsaturated fatty acids. PPARs (PPARα, PPARδ, and PPARγ) stimulate lipid and glucose utilization by increasing mitochondrial function and fatty acid desaturation pathways (Montaigne et al., 2021). Therefore, we speculated that arachidonic acid might be an activator of PPARδ. About downstream Fabp4, it encoded fatty acid-binding protein 4 controlled by most notably PPARγ, PPARδ, and FFA (Haunerland and Spener, 2004). Fabp4 bind long-chain FFA and are specifically expressed in adipocyte (Thompson et al., 2018). Although the underlying molecular mechanisms of Fabp4 expression and activity have not been fully elucidated, Omega-3 fatty acids were reported to decrease the expression and consecutive secretion of Fabp4, which had a positive effect on anti-obesity and reversal of insulin resistance (Furuhashi et al., 2016; Chung et al., 2021). Based on above, we supposed that the Fads1/PPARδ/Fabp4 signaling axis might be a direct target pathway of TF3 to reduce lipid accumulation, providing a more theoretical basis for further drug development and clinical application.

Apart from signaling axis-related genes, TF3 also regulated some other gene expressions related to NAFLD. Plin4, Lpl, and Acadm were also critical target genes of PPARs in the PPAR signaling pathway. Their expression decreased after TF3 treatment. With the progression of NAFLD, the Lpl/Fabp4/Cpt1 molecule axis and controlled fatty acid metabolism were generally upregulated since the NASH phase (Yang et al., 2021). Hence, Plin4, Lpl, and Acadm might produce beneficial effects on preventing the progression of NAFLD to nonalcoholic steatohepatitis. In addition, the increased expression of Fdft1, Tm7sf2, Ebp, and Dhcr7, involved in cholesterol biosynthesis, might indirectly promote primary bile acid biosynthesis. Bile acid supplementations and agonists for specific targets played an important role in decreasing lipid accumulation and treating metabolic liver disorders (Liu H. et al., 2016; Evangelakos et al., 2021). For example, obeticholic acid, the steroidal agonist of farnesoid X receptor, became the most promising drug for MAFLD/steatohepatitis (Younossi et al., 2019). These genes might exert a synergistic effect with the Fads1/PPARδ/Fabp4 signaling axis in TF3.

The gut microbiota plays a crucial role in NAFLD mitigation through various mechanisms such as energy absorption and storage, promoting insulin resistance and choline deficiency and interfering with bile acid metabolism (Blaut, 2015; Wang et al., 2020). Hence, we analyzed the changes of the gut microbiota in NAFLD with TF3 supplement and found that the community richness and diversity of the gut microbiota increased. This is similar to the infusions of green tea, oolong tea, and black tea (Liu Z. et al., 2016); in addition, another tea polyphenol (epigallocatechin-3-gallate) treatment (Ushiroda et al., 2019) suppressed fatty liver disease by improving gut dysbiosis or increasing the diversity of the gut microbiota or altered its structure in obese mice. Our analysis demonstrated that *Prevotellaceae_UCG-001*, *norank_f__Ruminococcaceae* and *GCA-900066575* were significantly increased and *Parvibacter* was decreased in the H-TF3 group compared with those in the ob/ob group at the genus level. *Ruminococcaceae* are butyrate-producing bacteria. Butyrate is a kind of short-chain fatty acid (SCFA) produced from resistant starch, dietary fiber, and low-digestible polysaccharides by the microbiota in the colon and distal small intestine via fermentation (Kau et al., 2011; Zhou et al., 2017). Butyric acid protected against HFD-induced hepatic steatosis, inflammation, and liver injury (Zhou et al., 2017). *Prevotellaceae_UCG-001* also produces SCFAs, which stimulates glucagon secretion and increases satiety to regulate fat synthesis and cholesterol in the liver and inhibit weight gain (Maljaars et al., 2008; Wang M. et al., 2019). Previous studies had shown that high-fat diet–induced obese mice had a higher abundance of *GCA-900066575* than wild mice (Li et al., 2020; Hu Q. et al., 2021). However, we found that the abundance of *GCA-900066575* increased in the H-TF3 group rather than in ob/ob obese mice. A study also reported that α-linolenic acid could alleviate fatty liver and increase the abundance of *GCA-900066575* (Gao et al., 2020). *Parvibacter* is a beneficial bacterium, but its abundance reduced rather than increased, as expected, in the TF3 group (Li et al., 2022). TF3 mainly increased the abundance of SCF-producing bacteria to synergistically reduce fat accumulation and hepatic steatosis. It is worth mentioning that we found that TF3 could modulate gut microbiota composition *in vivo* even by intraperitoneal injection.

Accumulating evidence has pointed out the importance of the gut–liver axis in the development of liver disease. According to the correlation between the gut microbiota and candidate genes involved in lipid metabolism and PPAR signaling pathways, biochemical parameters, we supposed that the changes of the gut microbiota might be related to the Fads1/PPARδ/Fabp4 signaling axis. They further decreased lipid levels and liver injury together in NAFLD with TF3 supplement. However, the mechanism is still unclear. Their interaction requires further research. Bile acid metabolism, gut microbial metabolites such as lipopolysaccharide (LPS), and gut barrier dysfunction contribute to chronic liver disease by abnormal regulation of the gut–liver axis (Schneider et al., 2018; An et al., 2022). In a subsequent study, we will deeply explore the mechanism of the gut–liver axis in TF3, clarify the pharmacological effects, and provide more detailed clinical data for the drug development of TF3 in preventing and curing NAFLD.

## Conclusion

It was demonstrated that TF3 was a safe and effective pharmacological ingredient for relieving NAFLD in the ob/ob mice model. The weight and waistline, fat accumulation, serum lipid, liver injury, and hepatic TG were alleviated by TF3 treatment. The beneficial effect of TF3 on NAFLD might be related to lipid metabolism regulated by the Fads1/PPARδ/Fabp4 axis and gut microbiota. The Spearman correlation indicated that the gut microbiota might associate with the hepatic Fads1/PPARδ/Fabp4 signaling axis. Their interaction requires further research. TF3 might be a promising drug for clinical use to improve NAFLD.

## Data Availability

The datasets presented in this study can be found in online repositories. The names of the repository/repositories and accession number(s) can be found in the following: https://www.ncbi.nlm.nih.gov/, PRJNA824024; https://www.ncbi.nlm.nih.gov/, PRJNA824955.
