# Supplementary material for: Effect of theaflavin-3,3′-digallate on leptin-deficient induced nonalcoholic fatty liver disease might be related to lipid metabolism regulated by the Fads1/PPARδ/Fabp4 axis and gut microbiota
Source: Front Pharmacol. 2022 Aug 29;13:925264. doi: 10.3389/fphar.2022.925264 (PMC9464872; doi:10.3389/fphar.2022.925264)
Supplement: Supplementary file 1 [file Table1.docx]

Table 1 RNA Sequencing Data Analysis

| Sample | Raw reads | Clean reads (%) | Error rate (%) | | Q20 (%) | Q30 (%) | GC content (%) | Total mapped |
| --- | --- | --- | --- | --- | --- | --- | --- | --- |
| WT1 | 51547906 | 51160430(99.25%) | 0.0239 | 98.47 | | 95.29 | 49.36 | 49566613(96.88%) |
| WT2 | 55837996 | 55466626(99.33%) | 0.0237 | 98.54 | | 95.45 | 49.94 | 53399611(96.27%) |
| WT3 | 46999630 | 46639770(99.23%) | 0.0236 | 98.57 | | 95.57 | 50.38 | 45071096(96.64%) |
| obob1 | 46006240 | 45726252(99.39%) | 0.0235 | 98.60 | | 95.65 | 49.60 | 44020714(96.27%) |
| obob2 | 49338542 | 49061926(99.44%) | 0.0236 | 98.57 | | 95.55 | 49.24 | 47561534(96.94%) |
| obob3 | 51593974 | 51251024(99.34%) | 0.0237 | 98.54 | | 95.45 | 48.10 | 49448967(96.48%) |
| H-TF31 | 59692984 | 59257088(99.27%) | 0.0235 | 98.59 | | 95.64 | 50.81 | 57454419(96.96%) |
| H-TF32 | 49104724 | 48689432(99.15%) | 0.0239 | 98.46 | | 95.31 | 48.74 | 47247392(97.04%) |
| H-TF33 | 50391932 | 50081270(99.38%) | 0.0235 | 98.61 | | 95.66 | 50.32 | 48674351(97.19%) |
